# Sonochemistry in light of third reactivity paradigm^☆^

**DOI:** 10.1016/j.ultsonch.2025.107523

**Published:** 2025-08-21

**Authors:** Sergey I. Nikitenko

**Affiliations:** ICSM, Univ Montpellier, UMR 5257, CEA, CNRS, ENSCM, Marcoule, France

## Abstract

Large number of chemical reactions can be described rigorously using classical thermodynamics and classical kinetics. However, there are an increasing number of examples of chemical reactions that deviate from “classical” behavior. Describing them requires considering quantum effects. The purpose of this review is to emphasize the importance of such “non-classical” reactions in sonochemistry. Quantum effects in sonochemistry are a direct consequence of the formation of nonequilibrium plasma inside collapsing bubbles. Spectroscopic studies of multibubble sonoluminescence revealed that intrabubble processes cannot be described by a single gas temperature. Rather, vibrational excitation and ionization must also be considered. Most clearly, quantum effects in sonochemistry, like those in “classical” chemistry, appeared for kinetic isotope effects, KIE. The anomalous H/D KIE during water sonolysis in the presence of noble gases can be understood in terms of electron quantum tunneling during the heterolytic splitting of a water molecule. In addition, the inverse ^13^C/^12^C KIE observed during water sonolysis in the presence of CO indicated a similarity with a non-equilibrium plasma generated by CO excitation in a gas phase. This KIE originated from the quantum vibration–vibration pumping mechanism. In the concluding part of the review, some perspective research directions are discussed.

## Highlights

• MBSL spectroscopy revealed formation of nonequilibrium plasma inside cavitation bubbles.
• Sonochemical plasma cannot be described by gas temperature; vibrational excitation and ionization must also be considered.
• H/D KIE revealed a strong contribution of electron tunneling in the process of water sonolysis.
• Invers 13C/12C KIE in sonochemical reactions of CO originated from plasma chemical vibration-vibration pumping mechanism.

## 1 Introduction

Kuhn defined a scientific paradigm as: “universally recognized scientific achievements that, for a time, provide model problems and solutions for a community of practitioners” [1]. The first and second reactivity paradigms of chemical reactivity are commonly considered in terms of thermodynamic and kinetic control respectively. The term ”third reactivity paradigm“ has recently been introduced by Schreiner to highlight the impotence of quantum effects, and in particular quantum tunneling, to control chemical reactions [2]. In fact, quantum tunneling is relevant when the de Broglie wavelength of the particle is of the order of the energetic barrier width. This is an obvious case for electrons. Nevertheless, the probability of quantum tunneling decreases sharply with increasing particle mass and particle kinetic energy, or in other words, with increasing temperature of the reacting system. From this point of view, it is difficult to expect the observation of quantum effects in sonochemical reactions, suggesting that sonochemical reactivity is driven solely by thermal homolytic cleavage of chemical bonds. However, the situation becomes different when ionized gas (plasma) is formed inside the collapsing bubble because the quantum tunneling of electrons can be observed even at high temperatures. In addition, some specific quantum effects with vibrationally exited molecules can be observed in nonequilibrium plasmas [3]. Therefore, this review will first focus on the experimental evidence for plasma formation during acoustic cavitation and then on the experimental observations of quantum phenomena associated with sonochemical plasma.

## 2 Spectroscopic observations of sonochemical plasma

In 1933 Marinesco and Trillat accidentally observed the darkening of photographic plates submitted to ultrasound in water [4]. They attributed this finding to the ultrasonic acceleration of Ag^+^ ions chemical reduction at the surface of plates. However, one year later Frenzel and Schultes [5] showed that photographic plate darkening is due to the light emission from sonicated water rather than from chemical reaction. Since then, hundreds of scientific papers have been devoted to the study of this fascinating phenomenon called sonoluminescence. In fact, two different types of SL can be distinguished: light emission from a cloud of bubbles (multibubble sonoluminescence, MBSL) and light emission from a single cavitation bubble trapped in a standing acoustic wave of relatively low acoustic pressure (single bubble sonoluminescence, SBSL).

For the first time SBSL in water was reported by Yosioka and Omura in 1962 [6]. Eight years later Temple described the same phenomenon in his MS thesis [7,8]. However, both observations did not attract the attention of the scientific community and only in 1990 Gaitan and Crum independently re-discovered and studied SBSL in water/glycerol mixtures [9]. In degassed water, the SBSL was bright enough to be visible to the naked eye. The experiments demonstrated the unique properties of this system: the light emission occurs every acoustic cycle at the final stage of collapse and the pulse duration of the light flash is below 200 ps [[10], [11], [12]]. SBSL spectra of degassed water showed no structures as well-defined lines and bands. Early SBSL studies attributed these spectra to blackbody emission, consistent with an adiabatic heating model. However, further reports by Gompf at al. [13], Hiller et al. [14] and Moran et al. [15] found no significant difference between the pulse width in the UV part of the SBSL spectrum (300–400 nm) and in the red part (590–650 nm). This contradicts a thermal model, which suggests that the red pulse of the blackbody emission should be about twice as long as the UV part. Moreover, the intensity of the pulse predicted by the blackbody model is about two orders of magnitude larger than the experimental values for the experimental parameters studied [16]. Several researchers suggested that the featureless SBSL spectra originated from bremsstrahlung rather than from blackbody emission [8,16]. Bremsstrahlung emission produced by the deceleration of an electron by an atomic nucleus in plasma. Bernstein et al. attributed a bright continuum in SBSL spectra to the emission from confined electrons because the Einstein A coefficient, which related to the rate of spontaneous emission of light, is ≈ 700 times larger for electron than for OH(A-X) molecular emission [17]. Alternatively, Lavrov attributed a continuum emission in SBSL spectra in water preequilibrated with Ar to the radiative dissociation of electronically excited H_2_* (a^3^ $\Sigma_{g}^{+})$ molecules and/or ArH* (A^2^Σ) excimers [18]. It should be noted that this hypothesis is not in conflict with the plasma model of SBSL.

**Fig. 1:**
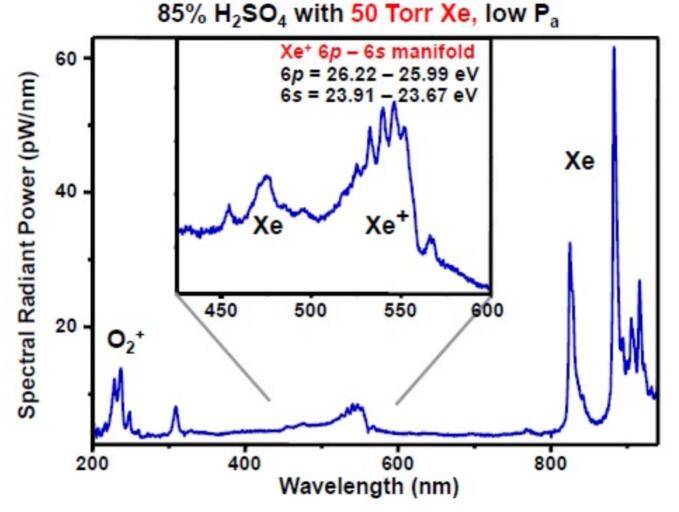
SBSL spectra from 85 % H_2_SO_4_ pre-equilibrated with 50 torr of Xe at the acoustic pressure P_a_ = 1.7 bar. []

Reproduced from 19.

The first direct observation of plasma in single cavitation bubble was reported by Flannigan and Suslick in concentrated H_2_SO_4_ pre-equilibrated with noble gases at the driven frequency of 20 kHz [19]. The SBSL spectrum collected in the presence of xenon (Fig. 1) shows a light emission from electronically excited and ionized Xe atoms as well as ionized oxygen O_2_^+^. Formation of O_2_^+^ species is inconsistent with any thermal process because the ionization energy of O_2_ molecule is more than twice its bond dissociation energy. Therefore, strong heat should lead to O_2_ homolytic dissociation rather than to ionization. On the other hand, the O_2_^+^ species can be formed by electron impact in plasma [3]. Furthermore, the SBSL spectra H_2_SO_4_ in the presence of noble gases show emission line from Xe^+^, Kr^+^ and Ar^+^ with the energies ranging from 26.0 eV to 34.2 eV [19]. On the other hand, the effective gas temperature calculated from Ar* line widths presuming thermal equilibrium inside the bubble is only about 1 eV (ca. 11000 K). Such a discrepancy cannot be understood by presuming only adiabatic heating during bubble collapse. The time-resolved spectra of SBSL in H_2_SO_4_ pre-equilibrated with Kr measured using streak camera revealed the spectrum evolution with time of collapse [20]. At 0.5 ns the SBSL spectrum exhibits line emission from excited krypton atoms, Kr*, centered at 810 nm (5s-5p transition). With evolution of time, the lines of Kr* disappear, the central wavelength moves from infrared to ultraviolet monotonously, and at 8.5 ns the spectrum shows featureless emission in the UV range. Emission from Kr* (E ∼ 9.9 eV) at the early stage of collapse is incompatible with the adiabatic heating model of SBSL. Interestingly, this observation is consistent with a hypothesis explaining continuum emission in SBSL spectra by radiative dissociation of noble gas-hydrogen excimer. The electronically excited atoms of noble gases can be formed by the electron impact of atoms in ground state and by cation-electron recombination via three-body collision mechanism [21].

**Fig. 2:**
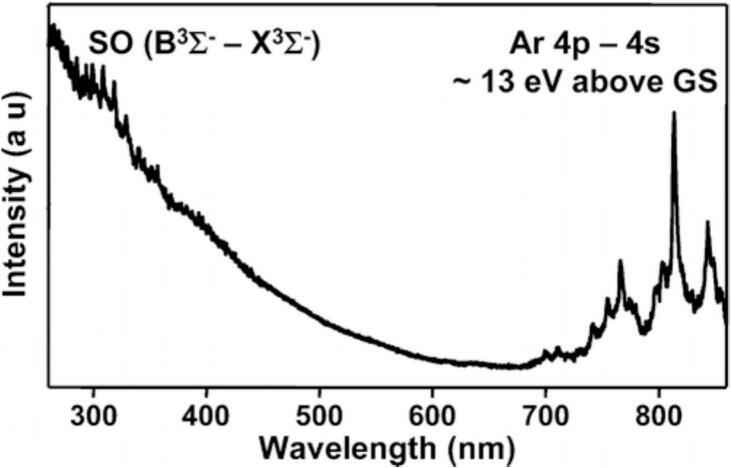
MBSL spectrum from 95 wt% H_2_SO_4_ in the presence of Ar, f = 20 kHz, P = 14 W cm^−2^, T = 298 K. []

Reproduced from 22.

Unlike SBSL, MBSL spectra in various solvents show molecular line emission over a wide range of conditions, but no emission bands from ionized species. In concentrated H_2_SO_4_ saturated with Ar, the MBSL spectrum shows emission from a broad continuum together with SO (B^3^Σ^−^ − X^3^Σ^−^) and 4p-4s excited Ar manifold (Fig. 2) [22]. An effective intrabubble temperature close to 8000 K was calculated from the thermalized fit of the Ar* emission band. As with the SBSL discussed above, this temperature is too low for the thermal excitation of an Ar atom, which requires approximately 13 eV. This indicates the absence of thermal equilibrium inside the collapsing bubble.

**Fig. 3:**
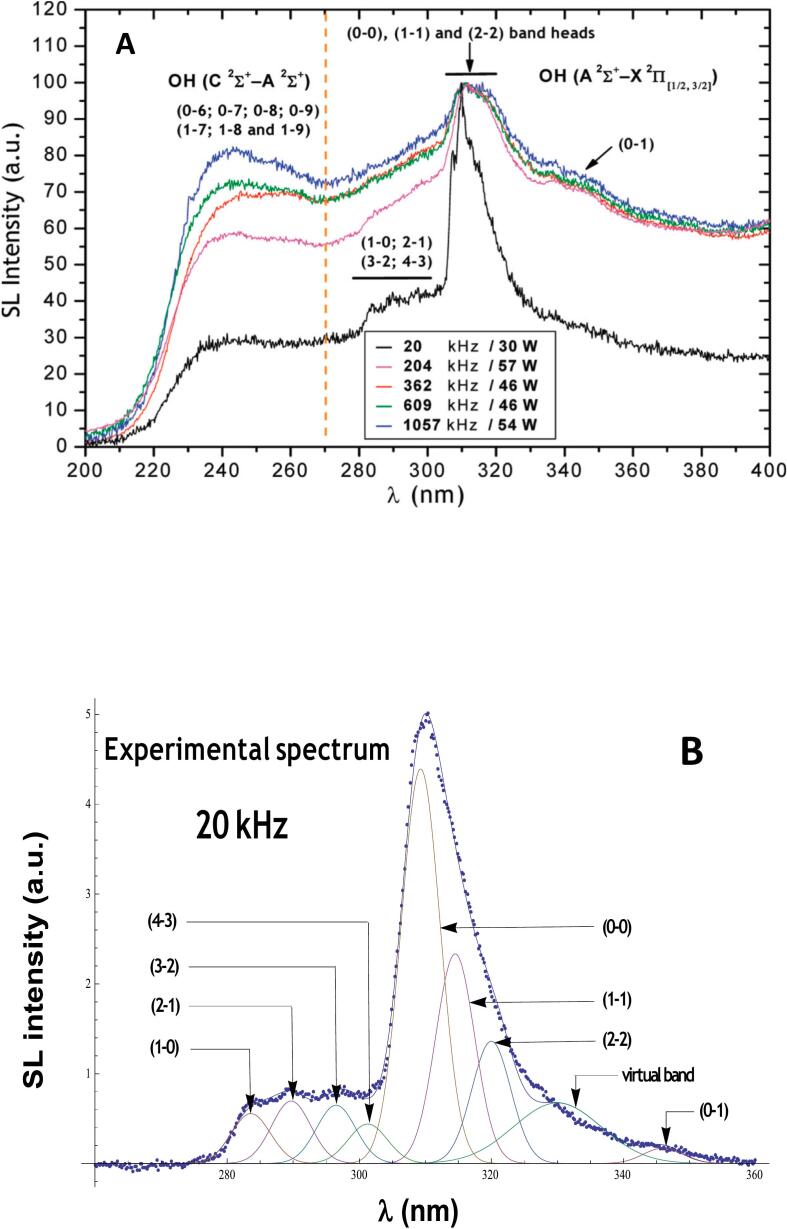
(A) Normalized MBSL spectra of water in the presence of Ar at 10 ± 1 °C, (B) Deconvoluted band of the OH(A-X) system at 20 kHz after the baseline subtraction. []

Reproduced from 26.

The MBSL spectra of pure water pre-equilibrated with Ar, Kr, and Xe are composed of the emission lines of excited OH^•^ radicals in A^2^Σ^+^ and C^2^Σ^+^ states and a broad continuum ranging from UV to near-infrared spectral range, which probably results from the superposition of several emission bands: H + OH^•^ recombination, water molecule de-excitation, and OH(B^2^Σ^+^-A^2^Σ) emission [[23], [24], [25], [26]]. Fig. 3A shows the MBSL spectra of excited OH^•^ radicals collected in water saturated with Ar and submitted to different ultrasonic frequencies [26]. Two spectroscopic systems can be distinguished: OH(A-X) system at 280–350 nm composed of (0–0), (0–1), (1–0), (1–1), (2–1), (2–2), (3–2), and (4–3) rovibronic transitions, and OH(C-A) system at 230–260 nm. The OH(C^2^Σ^+^) excited state originates from the electron impact on the H_2_O molecule clearly indicating the formation of a plasma inside the cavitation bubble [25]. The relative intensity of the OH(C-A) emission band increases with increasing ultrasound frequency, most likely, due to more ionized plasma formed at high frequency ultrasound. However, most of the rovibronic emission bands of OH(C-A) system are in far UV spectral range and, therefore, cannot be quantified in aqueous solutions. On the other hand, the deconvolution of the OH(A-X) band, as shown in Fig. 3B for 20 kHz ultrasound, offers the possibility to obtain the population of OH(A^2^Σ^+^) vibrational states and, consequently, to calculate the rotational (gas) temperature, T_r_, and the vibrational temperature, T_v_, of the excited OH^•^ radicals. The calculated T_r_ and T_v_ values of the OH(A^2^Σ^+^) excited state will be considered below in comparison with other spectroscopic probes.

**Fig. 4:**
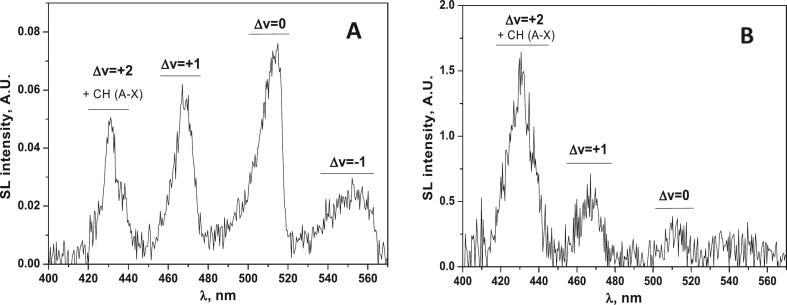
Swan band MBSL spectra with subtracted baselines of aqueous t-BuOH solutions in the presence of Ar, T ≈ 10 °C. (A) 20 kHz, 0.1 M t-BuOH, (B) 362 kHz, 8.5 10^−4^ M t-BuOH. []

Reproduced from 31.

It is worth noting that the MBSL spectra of some other radical species can also be used as a spectroscopic probe of intrabubble conditions. Suslick et al. observed the emission of excited C_2_* radical during ultrasonic treatment of some organic liquids [27,28] and diluted aqueous solutions of organic substrates submitted to 20 kHz ultrasound in the presence of Ar [29]. The intense emission bands from C_2_*, known as the Swan band (d^3^П_g_ → a^3^П_u_), are composed of several rovibronic transitions ranged from Δ*v* = −3 to Δ*v* = +2 [30]. The relative populations of Δ*v* levels are temperature sensitive and can be used for the calculation of T_r_ and T_v_ values [30]. Presuming temperature equilibrium inside the bubble (T_r_ ≈ T_v_) at 20 kHz, the authors [27,28] find an effective intrabubble temperature of 4500–5500 K. However, recent studies have shown that the ratio of Δ*v* = 0 and Δ*v* = +1 levels is strongly sensitive to the ultrasonic frequency (Fig. 4) and, in general, T_v_ > T_r_ [31].

**Fig. 5:**
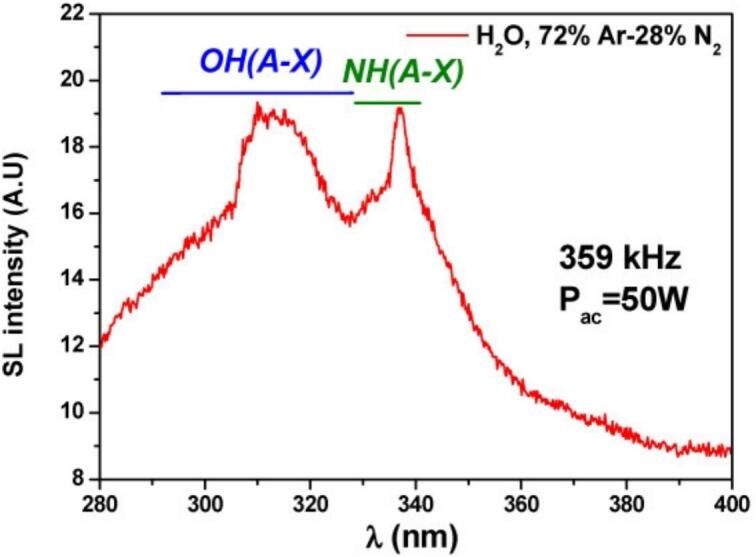
MBSL spectrum of water saturated with 72:28 Ar:N_2_ gas mixture subjected to 359 kHz ultrasound. T = 14 °C. []

Reproduced from 32.

The MBSL spectra collected at high ultrasonic frequency in water saturated with N_2_-Ar gas mixtures show the emissions of OH(A^2^Σ^+^ − X^2^П_i_) and NH(A^3^П – X^3^Σ^−^) systems (Fig. 5) [32]. It is worth noting that at 20 kHz solely the emission of OH(A-X) system is observed, which can be ascribed to less drastic conditions generated within the bubbles at 20 kHz ultrasound. Surprisingly, the emission bands of N_2_(C^3^П_u_ – B^3^П_g_) system at 337–391 nm is not observed even at high-frequency ultrasound. In general, vibrationally excited state N_2_(C^3^П_u_) is formed in plasma as a precursor of N_2_ molecule splitting [33]. However, in the presence of hydrogen atoms formed as a product of water sonolysis this state can be effectively quenched with the formation of excited NH(A^3^П) radical [34,35]:

(1) H_2_O−))) → H + OH^•^

(2) N_2_−))) → N_2_(C^3^П_u_)

(3) N_2_(C^3^П_u_) + H → NH((A^3^П) + N

Indeed, the N_2_(C^3^П_u_ – B^3^П_g_) emission band is observed during SBSL in nonaqueous H_2_SO_4_ in the presence of an air-Ar gaseous mixture [36].

**Fig. 6:**
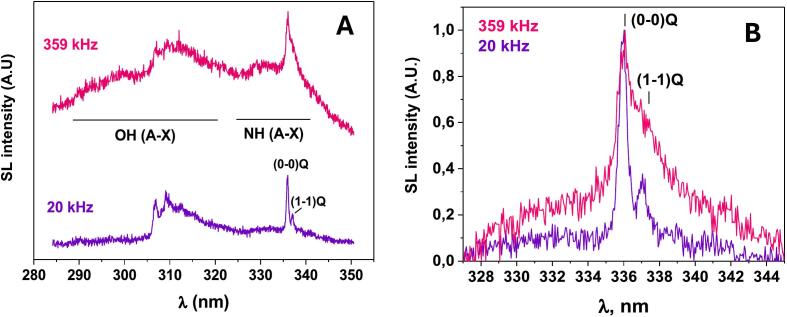
(A) MBSL spectra of 0.1 M NH_3_·H_2_O solutions at 359 kHz and 20 kHz in the presence of Ar. T = 14 °C, (B) Comparison of NH(A^3^П – X^3^Σ^−^) bands at 20 kHz and 359 kHz. The spectra were normalized to (0–0)Q line intensity after baseline subtraction. []

Reproduced from 37.

The intense NH(A^3^П – X^3^Σ^−^) emission band composed of (0–0)Q and (1–1)Q rovibronic transitions is also observed together with the OH(A^2^Σ^+^ − X^2^П_i_) emission during sonication of aqueous ammonia solutions saturated with Ar [37]. Fig. 6 shows that, like an OH(A-X) band, the shape of the NH(A-X) band strongly depends on the ultrasonic frequency. At 20 kHz, the shape of NH(A-X) band is similar to that reported during the dissociative excitation of NH_3_ molecule by collision with electronically excited Ar(^3^P_2,0_) atom in plasma generated by electric discharge [38]. However, the MBSL spectrum of NH(A-X) at high ultrasonic frequency shows stronger relative intensity of (1–1)Q transition indicating stronger vibrational excitation.

Table 1 summarizes the T_r_ and T_v_ values calculated for different systems using Specair software which enables rovibronic temperature determination in plasmas under conditions ranging from thermal equilibrium to thermochemical nonequilibrium [39]. For the OH(A) and NH(A) states, the T_v_ values increased with the ultrasonic frequency. Furthermore, the difference between T_v_ and T_r_, or, in other words, the deviation from thermal equilibrium, grows as the frequency rises. However, these tendencies are less apparent for C_2_* radicals, as these species form as a result of multiple interactions within the bubble, bringing them closer to thermal equilibrium. The observed inequivalence between T_v_ and T_r_ is typical for weakly ionized plasmas: T_e_ > T_v_ > T_r_ ≈ T_g_, where T_e_ is an electron temperature [3]. The T_e_ represents the average energy of electrons in plasma. In many nonequilibrium plasma systems, the T_e_ is about 1 eV (about 11,000 K). In general, the T_e_ values obey the Maxwellian distribution [3]. This means that, at T_e_ ≈ 1 eV, approximately 10 % of electrons have much higher energies close to 10 eV. Therefore, noble gas atoms can be excited and ionized in weakly ionized plasma, as observed by SBSL in sulfuric acid. On the other hand, the MBSL spectroscopic determination of T_e_ in aqueous solutions is difficult due to the lack of emission bands from ionized species or the H_α_ and H_β_ atomic states. Ndiaye et al. estimated the T_e_ values for sonochemical plasma produced by multibubble cavitation in water saturated with Ar using the spectral analysis of OH(A^2^Σ^+^-X^2^П_i_) rovibronic bands [26]. Their approach involved the calculation of OH(A-X) synthetic spectrum and its optimization by variation of T_e_ and T_v_ values presuming that the rotational population of the upper level is significantly the same as that of the ground state, and that the excitation of the electronic upper level is mainly due to electron impact. This non-direct method gives the T_e_ value about 0.7 eV at 20 kHz and about 1 eV at 1057 kHz.

**Table 1:** T_v_ and T_r_ values calculated by fitting the experimental MBSL spectra using Specair software of aqueous solutions saturated with Ar and submitted to power ultrasound. In general, the rotational temperature (T_v_), is considered equal to the gas temperature (T_g_) [3].

Solution	Ultrasonic frequency	Spectroscopic probe	T_r_ = T_g_, K	T_v_, K	T_v_ – T_r_, K	Reference
H_2_O	20 kHz	OH(A^2^Σ^+^ − X^2^П_i_)	5250 ± 250	5750 ± 250	500	[40]
H_2_O	100 kHz	OH(A^2^Σ^+^ − X^2^П_i_)	4750 ± 250	6500 ± 500	1750	[40]
H_2_O	362 kHz	OH(A^2^Σ^+^ − X^2^П_i_)	5250 ± 250	8000 ± 1000	2750	[40]
0.1 M NH_3_·H_2_O	20 kHz	NH(A^3^П − X^3^Σ^−^)	4000 ± 500	7000 ± 1000	3000	[37]
0.1 M NH_3_·H_2_O	359 kHz	NH(A^3^П − X^3^Σ^−^)	2200 ± 500	10000 ± 1000	7800	[37]
0.05–0.3 M t-BuOH	20 kHz	C_2_(d^3^П_g_ → a^3^П_u_)	4800 ± 1000	6300 ± 500	1500	[31]
4 10^−3^-1 10^−2^ M t-BuOH	204 kHz	C_2_(d^3^П_g_ → a^3^П_u_)	5800 ± 1000	5800 ± 500	0	[31]
(3.2–7.5) 10^−3^ M	362 kHz	C_2_(d^3^П_g_ → a^3^П_u_)	4000 ± 1000	8000 ± 500	4000	[31]
(2–5) 10^−3^ M	613 kHz	C_2_(d^3^П_g_ → a^3^П_u_)	4000 ± 1000	5000 ± 500	1000	[31]

## 3 Searching for solvated electrons

Spectroscopic observations of the formation of nonequilibrium plasma during multibubble cavitation raised questions about the formation of solvated electrons in solutions subjected to power ultrasound. In aqueous solutions, hydrated electrons can be formed in two different ways: either as primary species during ionization inside the bubble, or as secondary products in alkaline media, where the H^•^ atoms escaped from collapsing bubble react with hydroxyl anion OH^–^ yielding hydrated electrons. In early studies, several research groups used different sonochemical reactions as a probe of hydrated electrons. Anbar and Pecht reported formation of HD in the gas phase during sonolysis of deuterated formate anions at f = 800 kHz in the presence of Ar [41]. They suggested that HD can be formed by the reaction of deuterated formic acid with hydrated electron inside the bubble. However, it was concluded that the hydrated electrons are not formed because the yield of HD decreases with increasing solution acidity. Haïssinsky and Klein studied the sonochemical reaction (f = 960 kHz, Ar) of an aqueous mixture containing NaNO_3_ as a hydrated electron scavenger and NaCOOH as an OH^•^ radical scavenger [42]. They did not observe the formation of nitrite ions and concluded that hydrated electrons are absent in sonochemical systems. It is worth noting that authors [41,42] only considered radical reactions by analogy with radiolysis. However, more recent studies have revealed that thermal effects significantly contribute to the sonolysis of nitrate and formate ions. For example, thermolysis of nitrate ions may produce nitrite ions [43], and formate ions may produce hydrogen and a formyl radical as an intermediate [44]. Formyl radical is known to react rapidly with nitrite ion [45]. Therefore, strictly speaking, these results cannot be used as evidence that hydrated electrons are absent in sonochemistry.

Other researchers used metallic ions as electron scavengers and spin traps to probe hydrated electrons formation. It should be noted that the use of spin traps in sonochemistry has encountered difficulties due to their partial pyrolysis at the bubble/solution interface [46,47]. Guttiérrez et al. have demonstrated the sonochemical reduction of Tl^+^ cation by hydrated electrons at pH ≥ 12.7 (f = 1 MHz, 20 %H_2_/80 %Ar) formed due to the H^•^ atoms conversion in basic solutions [48]:

(4) H^•^ + OH^–^ ⇌ H_2_O + e_aq_^−^ (pK = 9.8)

Dharmarathne et al. reaffirmed these results using Fe(CN)_6_^3−^ and methyl viologen as electron scavengers in NaOH solutions (f = 355 kHz, Ar) [49]. They found the local interfacial concentration of hydrated electrons about 1.5 10^−3^ M and the hydrated electron lifetime less than 60 ns.

Recent studies of sonoluminescence spectroscopy have provided new insights into the formation of solvated electrons in sonochemical processes. Sharipov et al. reported the light emission from electronically excited complex *Ru(bpy)_3_^2+^ during multibubble sonication of neutral aqueous solutions of Ru(bpy)_3_^3+^ complex (20 kHz, Ar) [50]. The reduction of Ru(III) was attributed to the reaction with e_aq_^−^ escaped from collapsing cavitation bubble. It should be noted, however, that in a neutral aqueous solution, this reaction may compete with the reduction of Ru(III) by H^•^ atoms. Furthermore, a well-resolved emission band from electronically excited *Ce(III) ions was observed in SBSL spectra of moving single bubble collected in aqueous and ethylene glycol solutions of Ce(IV) (26 kHz, Ar) [51]. Interestingly, the formation of Ce(III) species in these systems can be explained solely by the reduction of Ce(IV) by e_aq_^−^ because H^•^ is unable to form electronically excited Ce(III) by the reduction of Ce(IV). The luminescence of electronically excited Tb(III)* initiated by a moving single bubble in aqueous solutions was found to be enhanced by scavengers of e_aq_^−^ (Cd^2+^, H^+^) [52]. It should be noted that Tb(III) ions are sonoexcited by collisions with hot particles at the bubble/solution interface, and the hydrated electrons are known to effectively quench Tb(III)*.

**Fig. 7:**
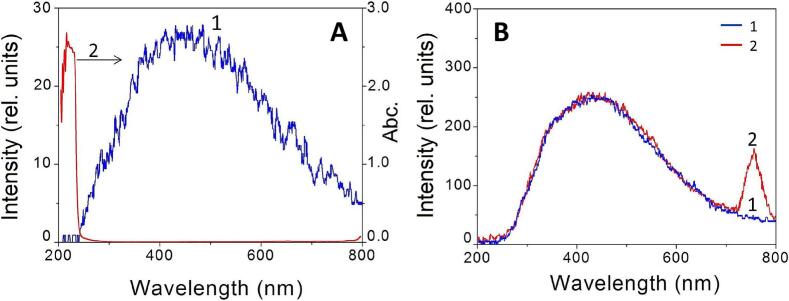
(A) 1. MBSL spectrum of liquid NH_3_ (20 kHz, T = −70 °C, Ar), 2. Absorption spectrum of liquid NH_3_. (B) SBSL spectra of moving single bubble in liquid NH_3_ (20 kHz, T = −70 °C, Ar). 1. Liquid NH_3_ without additives, 2. 10^−4^ M of SmCl_3_ dissolved in liquid NH_3_. []

The sonoluminescence spectra in liquid ammonia (20 kHz, Ar, −70 °C) reveal the formation of sonochemical plasma for both moving single-bubble and multibubble cavitation [53]. The physico-chemical parameters of NH_3_ listed in Table 2 show that its γ ratio is close to that of H_2_O. On the other hand, the thermal conductivity of NH_3_ is similar to that of Ar, but much lower than that of H_2_O. Finally, ammonia's ionization potential, E_i_, is much lower than those of argon and water, making it a favorable solvent for sonochemical plasma formation. The MBSL spectrum of liquid ammonia at −70 °C in the presence of Ar (Fig. 7A) exhibits a broad, structureless continuum across the spectral range from UV to near IR attributed to the glow-flash of the intrabubble plasma. The drastic decrease in sonoluminescence intensity at λ < 240 nm is due to ammonia self-adsorption. The SBSL spectrum of a moving single bubble in liquid NH_3_ resembles the MBSL spectrum, but its intensity is much higher (Fig. 7B). In a 10^−4^ M solution of SmCl_3_, the SBSL spectrum exhibits the emission band of the excited *Sm^2+^ at 755 nm (see spectrum 2 in Fig. 7B). This result is important because Sm^3+^ can be reduced to *Sm^2+^ by solvated electron, and not by H^•^ atom [54]. Therefore, the emission of *Sm^2+^ provides unequivocal evidence of the formation of solvated electrons during the sonolysis of liquid ammonia:

(5) NH_3_–))) NH_3_^+^ + e^−^ (inside the bubble)

(6) e^−^ + NH_3_ → e^−^_s_ (bubble/solution interface)

(7) e^−^_s_ + Sm^3+^ → *Sm^2+^

(8) *Sm^2+^ → Sm^2+^ + hν (755 nm)

**Table 2:** Some physico-chemical parameters of Ar, NH_3_, and H_2_O. The data were taken from NIST Chemistry WebBook, SRD 69, https://doi.org/10.18434/T4D303.

Parameter	Ar	NH_3_ (gas)	H_2_O (gas)
γ = C_p_/C_v_	1.67	1.38	1.33
Thermal conductivity W m^−1^ K^−1^	0.018	0.024	0.68
E_i_, eV	15.76	10.07	12.6

Reproduced from 53.

## 4 Kinetic isotope effects in sonochemistry

Observation of nonequilibrium plasma during acoustic cavitation through sonoluminescence spectroscopy suggests that the contribution of ionization to sonochemical reactions should not be neglected. However, in most cases, it is difficult to distinguish between heterolytic and homolytic sonochemical processes because of the relatively high gas temperature inside the collapsing bubble. In aqueous solutions, the H/D kinetic isotope effect (KIE) is commonly employed to clarify the reaction mechanisms [55]. Regarding the sonochemical water splitting, the H/D isotope separation factor α is calculated as $\alpha =\left(\frac{H_{2}}{D_{2}}\right)/{\left(\frac{H_{2}}{D_{2}}\right)}_{0},$ where ${\left(\frac{H_{2}}{D_{2}}\right)}_{0}$ is the initial isotopic composition of the H_2_O/D_2_O mixture and $\left(\frac{H_{2}}{D_{2}}\right)$ is the isotopic composition of the released hydrogen. Generally, there are two types of H/D KIE: solvent KIE and primary KIE [55,56]. The solvent H/D KIE refers to the difference in physicochemical properties between H_2_O and D_2_O, such as diffusion coefficient, viscosity, vapor pressure etc. However, these differences are quite small, and the α value of the solvent H/D KIE does not exceed 1.10 [56]. The semiclassical primary H/D KIE originates from the cleavage of chemical bonds. For the homolytic splitting of water molecule inside the cavitation bubble, it can be quantified as:

(9) $$\alpha =exp\left(\Delta E/RT_{g}\right)$$

where ΔE = 5.8 kJ·mol^−1^ is a difference between the zero-point energies of the ground states for O–H and O–D bonds and T_g_ is a gas temperature [55]. For a gas temperature of T_g_ = 5000 K inside a bubble produced by 20 kHz ultrasound in argon-saturated water, equation (8) yields an α value of 1.15. Mišik et al. studied the H/D KIE during sonolysis of an equimolar H_2_O/D_2_O mixture (f = 50 kHz, Ar) using H^•^ and D^•^ spin traps [46]. They found that the spin traps strongly influenced the α value due to their thermolysis at the bubble/solution interface. Using the most stable trap, N-*tert*-butyl-α-phenylnitrone (PBN), the value of α was determined to be 1.28 ± 0.08, which corresponds to T_g_ = 2500 K according to the equation (8). This value is much lower than the T_g_ measured by sonoluminescence spectroscopy. Moreover, this temperature is too low to provide thermal dissociation of water molecules inside the bubble [57]. This discrepancy may be attributed to the competition between H^•^ (D^•^) atoms scavenging with PBN and their recombination leading to molecular hydrogen release.

**Fig. 8:**
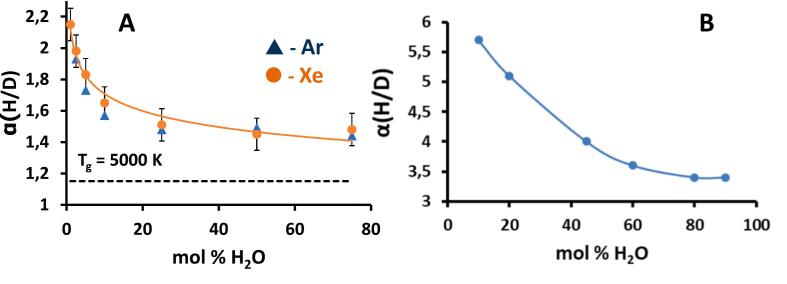
(A) Dependence of H/D KIE on H_2_O concentration in H_2_O/D_2_O mixtures saturated with Ar and Xe. f = 20 kHz, P_ac_ = 19 W, T = 10 °C. The dotted line shows calculated the semiclassical α value at T_g_ = 5000 K. Reproduced from [58]. (B) H/D KIE during water vapor γ-radiolysis, T = 160 °C. Reproduced from [59].

More recently, the H/D KIE was studied during H_2_O/D_2_O mixtures sonolysis (f = 20 kHz, Ar, Xe) using mass-spectrometric measurements of formed H_2_, D_2_, and HD species [58]. It was found that the released hydrogen is enriched with light isotope, regardless of the experimental conditions. Furthermore, the values of the α factor depicted in Fig. 8A revealed several striking features. First, the values of α are much larger than what is calculated assuming a thermal process at T_g_ = 5000 K. Additionally, the α values decrease as the H_2_O content increases, reaching a steady value close to α = 1.5. The α values are very similar for Ar and Xe, despite Xe-saturated water showing much higher overall sonochemical activity compared to Ar-saturated water. These results clearly demonstrate the significant role of non-thermal processes in the sonochemical water splitting mechanism. It is worth noting the distinct similarity in the dependence of the α factor on the H_2_O/D_2_O ratio for sonochemistry and γ-radiation (Fig. 8B). In radiation chemistry, the increase in α at a higher concentration of D_2_O is attributed to the slower decay of the solvated electron e_s_^−^ in D_2_O relative to H_2_O [60].

In general, the H^•^ atoms are produced during water radiolysis by two mechanisms: (i) dissociation of the covalent O-H bond of an excited H_2_O* molecule, which is formed from the recombination of electrons and H_2_O^+^ cations, and (ii) tunneling of electrons toward hydronium ions (H_3_O^+^) through the shell of water molecules surrounding both species, which is called electron conversion to hydrogen [61,62]. The homolytic splitting of the O-H bond obeys a semiclassical “zero-point energy” approach. On the other hand, isotope selectivity via the electron tunneling mechanism depends on the probability of electron tunneling, P_t_, through potential barriers of species with different isotopic composition. According to the Wentzel-Kramer-Brillouin equation, P_t_ is exponentially inversely proportional to the potential barrier width, *L*, and to the square root of the barrier height, *V* [63]. Therefore, even a small difference in either the *L* or *V* values would create large difference of P_t_ and, consequently, strong KIE. Note that both *L* and *V* values are larger for heavy isotopes. Hence, the electron quantum tunneling would be faster for lighter isotopes. In addition to the large H/D KIE reported for water vapor radiolysis [59], Muto et al. found extremely high α(H/D) values for radiolysis of frozen water, ranging from 10^2^ to 10^3^, presumably due to electron tunneling [61].

**Fig. 9:**
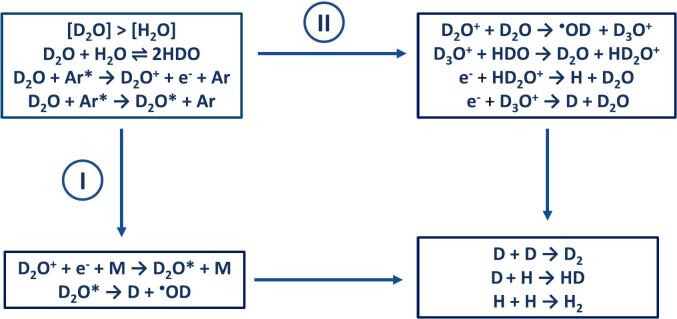
Suggested plasma chemical mechanism of the sonochemical water splitting. I is a homolytic reaction pathway, II is a heterolytic reaction pathway. For simplicity, a mixture in which [D_2_O] > [H_2_O] is considered. Under these conditions, D_2_O and HDO are the mixture's main components.

Similar to radiolysis, Fig. 9 illustrates the plasma chemical mechanism of sonochemical water splitting. Inside the collapsing bubble, collisions of water molecules with excited Ar*(Xe*) atoms can lead to their electronic excitation and ionization. The reaction pathway I represents homolytic splitting of excited water molecules, which should not lead to significant H/D KIE. On the other hand, the reaction pathway II shows a heterolytic water splitting mechanism involving hydrated electron/hydrogen conversion, which is limited by electron tunneling and accompanied by a strong H/D KIE. It is worth noting that the extremely short lifetime of the intrabubble plasma may kinetically inhibit thermal dissociation of H_2_O molecules. A recent time-correlated photon counting study of MBSL in water (20 kHz, Ar) revealed that the duration of continuum sonoluminescence pulses is approximately 1.8 ns [64]. On the other hand, the characteristic time for thermal water splitting is much longer, estimated at 0.1 µs for T_g_ = 5000 K and P = 500 bar. [65]. Therefore, only a small fraction of water inside the collapsing bubble can dissociate by thermal mechanism during the lifetime of sonochemical plasma. In contrast, the electron tunneling traversal time was found to be in the range of hundreds of attoseconds (10^−16^ s) [66], which favors a heterolytic mechanism.

**Fig. 10:**
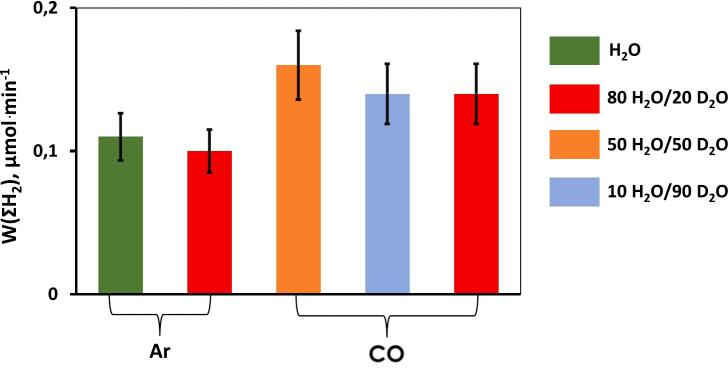
Rate of hydrogen formation, W(ƩH_2_), ΣH_2_ = H_2_ + HD + D_2_, during sonolysis of H_2_O/D_2_O mixtures in the presence of Ar and CO. f = 20 kHz, P_ac_ = 19 W, T = 20 °C. []

Reproduced from 67.

Another type of quantum effect was observed during the ultrasonic treatment of water (20 kHz) in the presence of pure carbon monoxide [67]. As discussed above regarding the sonochemical activity of ammonia, the significant sonochemical activity of water saturated by CO is also difficult to explain using the quasi-adiabatic heating model of cavitation. The specific heat ratio is lower for CO (γ = 1.40) than for Ar (γ = 1.67). In addition, the thermal conductivity of CO (25 mW m^−1^ K^−1^ at 300 K) is larger than that of Ar (17.9 mW m^−1^ K^−1^ at 300 K). Therefore, lower sonochemical activity is expected in the presence of CO than Ar. However, Fig. 10 shows that the hydrogen production rate in H_2_O/D_2_O mixtures, W(ΣH_2_), where ΣH_2_ = H_2_ + HD + D_2_, is higher with CO than with Ar. This striking result can be understood in terms of a plasma chemical approach because the ionization potential of CO (14.01 eV) is lower than that of Ar (15.76 eV), but similar to that of Kr (13.99 eV). The values of W(ƩH_2_) are practically independent on H_2_O/D_2_O ratio indicating weak solvent effect. In pure CO, the formation of H_2_O_2_ vanished due to the efficient scavenging of OH^•^ radicals with CO molecules.

**Fig. 11:**
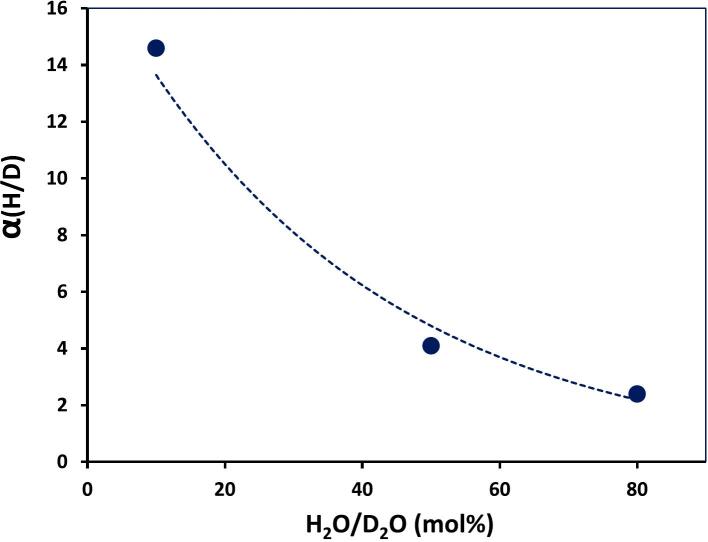
Dependence of H/D KIE on H_2_O concentration in H_2_O/D_2_O mixtures saturated with CO. f = 20 kHz, P_ac_ = 19 W, T = 20 °C. []

Reproduced from 67.

Surprisingly, the presence of CO strongly increases the H/D KIE up to α_H_ = 14.6 in the 10 %H_2_O/D_2_O mixture (Fig. 11) compared to that in Ar (α_H_ = 1.7, see Fig. 8). Such striking difference can be attributed to relatively low energy of CO molecule electronic excitation equal to 5.5 eV (A^1^Π) [3], which is lower than the energy of H_2_O molecule repulsive excitation state (7.5 eV, A^1^B_1_) [68]. This reduces the contribution of homolytic splitting of H_2_O molecules with low isotopic selectivity. Therefore, in the presence of CO, the observed H/D KIE is mostly to be due to a heterolytic process with high isotopic selectivity.

Ultrasonic treatment of water saturated with CO also results in the formation of CO_2_. The first source of CO_2_ in this system is the scavenging of OH^•^ radicals by CO molecules. In addition, authors [67,69] reported the formation of a carbonaceous solid product during the sonochemical process. This product has been identified as a mixture of disordered graphitic carbon and (C_3_O_2_)_x_ polymer. Such species are typically formed during CO disproportionation in plasma [3,70]:

(10) CO + e^−^ → CO* + e^−^

(11) CO* + CO* → C + CO_2_

(12) C + CO → C_2_O

(13) C_2_O + CO → C_3_O_2_

(14) xC_3_O_2_ → (C_3_O_2_)_x_

where CO* is a vibrationally excited CO molecule formed by electron impact. It was found that both products of the CO sonochemical disproportionation, CO_2_ [67] and carbonaceous species [69], are enriched with heavy carbon isotope ^13^C. The α_C_ value for CO_2_ is 1.26 ± 0.05, regardless of the H_2_O/D_2_O ratio. The inverse ^13^C/^12^C KIE during CO disproportionation, called Treanor effect, is a well-known phenomenon in nonequilibrium CO plasma produced by glow discharge or laser excitation [3,70]. This effect is based on the exchange of vibrational quanta between CO molecules, which leads to the non-Boltzmann distribution of excited CO molecules (V-V pumping) [70,71]:

(15) CO(ν) + CO(w) ⇌ CO(ν − 1) + CO(w + 1)

For T_v_ > T_g_, the value of α_C_ can be written as [3]:

(16) $$\alpha_{C}\approx exp\left[\frac{\Delta \omega }{\omega }E_{a}\left(\frac{1}{T_{g}}-\frac{1}{T_{v}}\right)\right]$$

where $\frac{\Delta \omega }{\omega }$ is the relative isotopic shift of oscillation frequency and *E_a_* is the activation energy of CO disproportionation. According to this equation, heavy isotopes would react faster due to higher T_v_ values. The ^13^C/^12^C isotopic selectivity reported for sonochemistry is lower than for non-equilibrium plasmas generated by electric discharge in CO gas where the *α_C_* value can reach 2 or 3 at near room *T_g_* temperature [3,72]. This difference can be attributed to the high gas temperature inside the bubble leading to a drop of α_C_ value according to equation (16). Using the values of $\frac{\Delta \omega }{\omega }=0.041$, *E_a_* = 6 eV, and *T_v_* = 5.5 eV (the highest vibrational temperature) of CO molecule available in the literature [3,73], as well as *T_g_* = 0.43 eV (T_g_ = 5000 K), the estimated *α_C_* value to be equal to 1.7, which is still larger than the experimental value. Such a discrepancy would most likely be due to the fact that the ^13^C-enriched CO_2_ originated from CO disproportionation is diluted by much less enriched CO_2_ formed by CO oxidation with the OH^•^ radical.

## 5 Concluding remarks

Spectroscopic studies of sonoluminescence revealed the formation of nonequilibrium plasma during the collapse of cavitation bubbles. Therefore, intrabubble processes cannot be described by a single gas temperature; vibrational excitation and ionization must also be taken into account. Studies have shown that MBSL spectra in aqueous solutions can be considered as a real fingerprint of sonochemical plasma, enabling the calculation of vibrational and gas temperatures inside bubbles at different ultrasonic frequencies and with different carrier gases. Significant line broadening in MBSL emission spectra over a long period has been attributed exclusively to high pressure at the final stage of collapse [74]. However, the extremely broad emission lines in the MBSL spectra in the presence of Xe could not be explained solely by high intrabubble pressure [25,37]. In addition, fitting the molecular lines of different radical species (OH, NH, C_2_, CN) observed during the sonication of aqueous solutions with 362 kHz ultrasound in the presence of Ar-based gas mixtures yielded the effective pressure values ranging from 1000 to 10000 bar [75]. These surprising results suggest that pressure is not the main cause of line broadening. In terms of plasma-chemical model of cavitation, line broadening can be explained by Stark effect. Stark broadening originates from the molecular emission lines perturbation by charged particles in plasmas [76]. While this effect is widely used to measure electron density in nonequilibrium plasmas, further spectroscopic studies of sonoluminescence are needed to establish a quantitative relationship between line broadening and sonochemical plasma electron density.

The finding of H/D and ^13^C/^12^C KIE in sonochemical reactions clearly demonstrate that quantum effects, such as electron quantum tunneling and V-V pumping, are important for understanding sonochemical mechanisms. Interestingly, the non-Boltzmann overpopulation of CO molecule vibrational states via V-V pumping, which leads to the ^13^C/^12^C KIE, has also been observed in MBSL spectra of OH^•^ radicals in water [26,77]. Currently, most research on KIE has focused on the 20 kHz range. It is worth noting that, in 1997, Dekerckheer et al. studied Weissler reaction in H_2_O and D_2_O subjected to 20 kHz and 1.7 MHz ultrasound in the presence of air [78]. They found a weak KIE at 20 kHz. In contrast, the reaction rate in H_2_O was nearly twice that in D_2_O at 1.7 MHz. In future studies, it would be interesting to investigate MBSL spectroscopy in combination with H/D KIE at different ultrasonic frequencies and in the presence of different carrier gases.

Another interesting area of research is a spectroscopic study of luminescence produced by hydrodynamic cavitation. Light emission of hydrodynamic cavitation is known since 1967 [79], but spectroscopic data on this phenomenon is still scarce. Recently, Biryukov et al. reported the emission spectrum of hydrodynamic cavitation in the presence of air/Ar mixture, consisting of emissions from OH(A^2^Σ^+^ − X^2^П_i_) and N_2_(C^3^Π – B^3^Π) systems, H_α_ (Balmer series) and excited Ar* atoms [80]. The analysis of molecular bands revealed strong deviation from thermal equilibrium: T_v_ = 4500 K and T_g_ = 300 K respectively. Very strong overpopulation of argon level with energy 13.48 eV (λ = 750.4 nm) was explained by the participation of argon in the hydrogen recombination:

(17) H^+^ + Ar + e^−^ → ArH^+^ + e^−^ → Ar* + H^•^

In general, the reported hydrodynamic emission spectrum differs greatly from the MBSL spectra in water generated by power ultrasound, indicating the different mechanisms of nonequilibrium plasma formation. The authors [80] suggested triboluminescent glow mechanism caused by the friction of liquid against the wall in the narrow part of the hydrodynamic channel. This suggestion aligns with a calculated gas temperature that is nearly equal to ambient bulk temperature. However, further spectroscopic studies are needed to bridge a gap between hydro- and sonoluminescence.

Coupling of sonoluminescence and sonochemistry with an external magnetic field can bring valuable information about the mechanisms of sonochemical processes. Young et al. found a quadratic dependence between the forcing pressure required for SBSL and a strong uniform magnetic field of the order of 5–20 T [81,82]. Similar damping effect of the magnetic field was reported for multibubble cavitation produced by focused ultrasound at 1.6 MHz [83] and for hydrodynamic cavitation in microchannels [84]. Meanwhile, the origin of cavitation dumping in strong magnetic fields remains a topic of debate. DiDonna et al. suggested that the magnetic fields influence gas solubility [82]. Yasui attributed this effect to the action of the Lorenz force on moving polar water molecules [85]. On the other hand, Bouzehouane et al. suggested that the influence of magnetic field arises from the magnetic induction and magnetic field gradient in diamagnetic fluids with low electrical conductivity [84]. Clearly, the magnetic field must be coupled with many hydrodynamic parameters that control bubble motion in order to change sonoluminescence by a factor of unity. In principle, the magnetic field could also influence the intrabubble conditions. From this perspective, it would be interesting to study how a magnetic field influences the sonoluminescence spectra and the H/D KIE during water sonolysis. Recently, it was demonstrated that applying a magnetic field can promote electron tunneling if the magnetic energy (ca. 10 T) exceeds the potential barrier height [86,87].

Finally, the recent observation of sonoluminescence in liquid ammonia provides an opportunity to study sonochemical reactions driven by solvated electrons. In water, solvated electrons hardly escape cavitation bubbles. However, the lifetime of e_s_^−^ in liquid ammonia is much larger than in water. This makes reactions involving ultrasonically generated e_s_^−^ more probable. Gareev et al. observed an emission band centered at 400 nm from excited (Ce^3+^)* ions in the sonoluminescence spectrum generated by a moving single bubble driven by 31.9 kHz ultrasound in solutions of Ce^4+^ sulfate in liquid ammonia [88]. In this process, the reduction of Ce^4+^ by e_s_^−^ escaped cavitation bubble yields excited (Ce^3+^)* ions, unlike the reduction of Ce^4+^ by H^•^ atoms leading to Ce^3+^ ions in a ground state. It is worth noting that liquid ammonia can be considered as a quantum fluid, wherein its pyramidal molecular structure inverts as the nitrogen atom changes position from one equilibrium point to another via the quantum tunneling at a rate of 2.4 10^12^ Hz [89]. In dilute lithium-ammonia solutions, the dominant mechanism of electron-solvent interaction results from an electron tunneling characterized by a variable donor–acceptor distance consistent with a rapidly fluctuating liquid structure [90]. These results demonstrate the significant potential of using liquid ammonia to observe specific sonochemical quantum effects. From a practical point of view, sonochemical splitting of ammonia could be an attractive method for hydrogen production. Liquid ammonia is considered as promising material for hydrogen storage and transportation [91]. Existing ammonia-to-hydrogen conversion technologies consist of ammonia evaporation and thermocatalytic degradation at approximately 400 °C [91]. Sonochemical degradation of ammonia can be performed in a liquid phase without evaporation and does not require a catalyst.

## CRediT authorship contribution statement

**Sergey I. Nikitenko:** Writing – review & editing, Writing – original draft, Methodology, Conceptualization.

## Declaration of competing interest

The author declare that they have no known competing financial interests or personal relationships that could have appeared to influence the work reported in this paper.
